# Analysis of Relationship between Electroencephalograms and Subjective Measurements for In-Vehicle Information System: A Preliminary Study

**DOI:** 10.3390/ijerph182212173

**Published:** 2021-11-19

**Authors:** Nahyeong Kim, Mungyeong Choe, Jaehyun Park, Jungchul Park, Hyun K. Kim, Jungyoon Kim, Muhammad Hussain, Suhwan Jung

**Affiliations:** 1 Department of Industrial & Management Engineering, Incheon National University (INU), Incheon 22012, Korea; kimnh@inu.ac.kr (N.K.); mgyeong@inu.ac.kr (M.C.); mhussain@inu.ac.kr (M.H.); suhwan94@inu.ac.kr (S.J.); 2 Department of Safety Engineering, Korea National University of Transportation, Chungju 27469, Korea; jcpark@ut.ac.kr; 3 School of Information Convergence, Kwangwoon University, Seoul 01897, Korea; hyunkkim@kw.ac.kr; 4 Department of Computer Science, Kent State University, Kent, OH 44240, USA; jkim78@kent.edu

**Keywords:** in-vehicle infotainment system (IVIS), display, usability, electroencephalogram (EEG), questionnaire

## Abstract

In this study, we explored the relationship between objective and subjective measures for usability evaluation in in-vehicle infotainment systems (IVISs). As a case study, four displays were evaluated based on cluster location and display orientation (that is, front–horizontal, front–vertical, right–horizontal, and right–vertical). Thirty-six participants performed tasks to manipulate the functions of the IVISs and data were collected through an electroencephalogram (EEG) sensor and questionnaire items. We analysed a model that estimated EEG-based objective indicators from subjective indicators. As a result, the objective indicators reflected the subjective indicators and were considered to explain the driver’s cognitive state. Although EEG data were collected from only four participants, this study proposed an experimental design that could be applied to the analysis of the relationship between the subject’s evaluation and EEG signals, as a preliminary study. We expect the experimental design and results of this study to be useful in analysing objective and subjective measures of usability evaluation.

## 1. Introduction

The in-vehicle display is an infotainment system that not only conveys necessary information to the driver but also provides pleasure. When considering the infotainment system, safety is a major concern because multitasking with secondary tasks during driving can cause traffic accidents. The major secondary tasks performed simultaneously with driving include checking driving information and manipulation of the audio, video, and navigation (AVN) systems [1,2]. There have been several studies aimed at minimizing negative effects by studying cognitive conditions using biological indicators. Strayer et al. [3] developed a systematic framework to evaluate driver distractions [4,5,6,7,8,9].

There have been many studies in which objective indicators were extracted and analysed based on the driver’s biological signs, for example, analyses of the driver’s condition using objective indicators based on electroencephalogram (EEG) signals [10,11,12]. In particular, Wang et al. [13] and Baldwin et al. [14] confirmed the driver’s continuous attention using a band power indicator. Additionally, studies have been conducted to propose different models for predicting driver sickness and fatigue [15,16], and deep-learning-based models for predicting cognitive conditions [17,18,19]. Studies have also been conducted to extract and analyse objective indicators based on eye-tracking data, together with usability evaluation of in-vehicle infotainment systems (IVISs) [20,21,22]. Kim et al. [23] analysed task performance according to the interface of the dashboard displays, using off-road glance time. In this study, the task completion time was calculated and the performance during an AVN operation was confirmed for various types of displays. Kula et al. [24] conducted a study to evaluate the usability of various cluster designs and IVISs with both objective and subjective measurements. They used biometric sensors such as EEGs for objective evaluation and a questionnaire for subjective measurements. A few other studies have also evaluated IVISs through subjective indicators [20,21]. Some of the major subjective indicators used in previous usability studies include interview-based data and various questionnaires such as the system usability scale (SUS), user experience questionnaire (UXQ), and NASA task load index (NASA-TLX). However, previous studies have been insufficient to analyse the relationship between objective and subjective indicators.

As drivers use IVISs more actively, automotive companies are focusing more on new designs and improvements to IVISs to make them more attractive to consumers. Recently, there has been a trend towards introducing a large display at the front of the vehicle [25]. In this regard, studies have been actively conducted to explore the effects of various design elements (for example, touch-key size and display position) of in-vehicle displays on driving [26,27,28]. In particular, Ma et al. [21] investigated the usability effects of touch-screen size (17, 10, 9, and 7″) and orientation (horizontal or vertical) of IVISs through eye tracking and questionnaire data, in terms of the objective and subjective indicators.

In this study, we analysed the relationship between the indicators for evaluating the usability of in-vehicle displays through modelling. In order to predict objective indicators using subjective indicators, we compared and evaluated several types of new in-vehicle displays as a case study, using metrics such as task completion time, performance-related psychophysiological indices, and questionnaire item scores. Thus, we evaluated whether the EEG-based objective indicator reflected the subjective indicators and explored its specific relevance, and the results of the indicators according to various displays were analysed. The contributions of this study are (1) an experimental design is proposed for analysing the relationship between objective and subjective indicators of usability evaluation using stepwise regression analysis, as a preliminary study, (2) the results of this study can be used as basic data for research on objective and subjective indicators and the relationship between them, and (3) the study can be used as a reference material in research into evaluating the usability of in-vehicle displays.

## 2. Methods

### 2.1. Participants

Thirty-six subjects, 20 males and 16 females with a mean age of 36.81 (±12.17) yr, participated in this study. Their average driving experience was 9.06 (±8.77) yr, with an average frequency of 3.69 (±3.19) h per week. The average driving distance of the participants was 111.11 (±119.90) km per week. The EEG data were collected from four males because only they voluntarily consented to allow measurement of their biosignals. Questionnaire data were collected from all participants. Participants signed an informed consent form and were free to withdraw their consent.

### 2.2. Apparatus

In this experiment, a driving simulator and two different sizes of monitors were used. The monitor sizes were 12.3” and 15.6”, and they were installed at the cluster and centre fascia locations, respectively. The EEG signal was measured using a multi-channel, wireless, portable Emotiv EPOC+ headset (Emotiv, San Francisco, CA, USA). Electrodes were placed according to the 10–10 system [29]. The locations of the electrodes were AF3, F7, F3, FC5, T7, P7, O1, O2, P8, T8, FC6, F4, F8, and AF4. EmotivPRO (version 2.0, Emotiv, San Francisco, CA, USA) software was used to analyse the EEG data. Raw EEG and performance-related psychophysiological indices were collected in real time at sampling rates of 128 and 2 Hz, respectively. Performance-related psychophysiological indices were analysed as built-in functions in the software using raw EEG signals.

### 2.3. Experimental Design and Task

**Figure 1:**
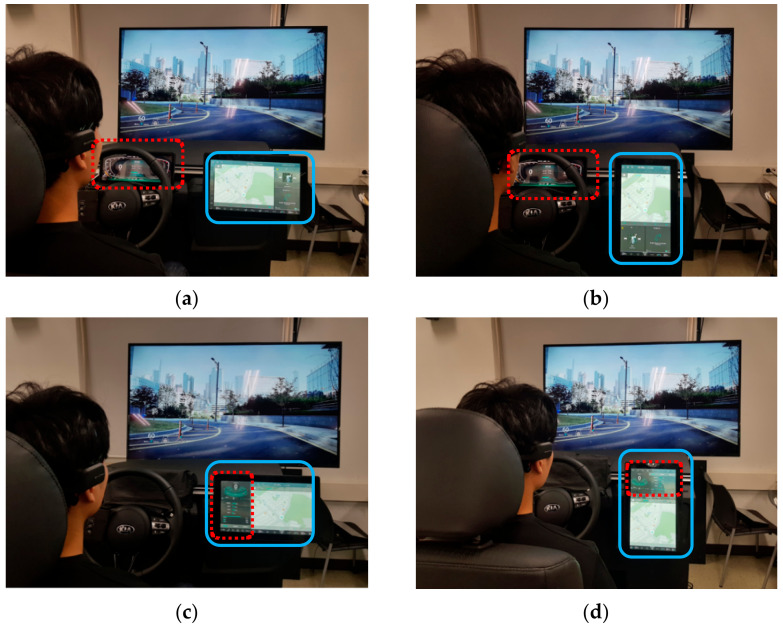
Types of displays: (**a**) front–horizontal; (**b**) front–vertical; (**c**) right–horizontal; (**d**) right–vertical.

Four experimental settings based on the cluster location (front or right side) and the main axis of the display (horizontal or vertical) were designed, as shown in Figure 1. The displays of the cluster are shown as dotted red boxes and the right-side displays (centre fascia monitor) are shown as blue boxes. In Figure 1a,b, the clusters are displayed on the front display monitor behind the driver’s steering wheel and the right-side displays of the centre fascia monitor are horizontal (Figure 1a,c) or vertical (Figure 1b,d). In Figure 1c,d, the clusters are displayed on the centre fascia monitor as dotted red boxes. The detailed combinations of the displays are shown in Table 1.

The experimental task was to manipulate vehicle functions and IVISs according to the given instructions (Table 2). Three ergonomic experts derived the tasks for this experiment by considering commonly used functions of current and future vehicle models through a systematic procedure. Initially, all possible functions related to IVISs were collected, and the most common functions were selected. After evaluating the importance of the conducted tasks, the final tasks were selected based on the functions used in both driving and non-driving conditions. The importance of orientation and cluster location was also considered during this stage.

**Figure 2:**
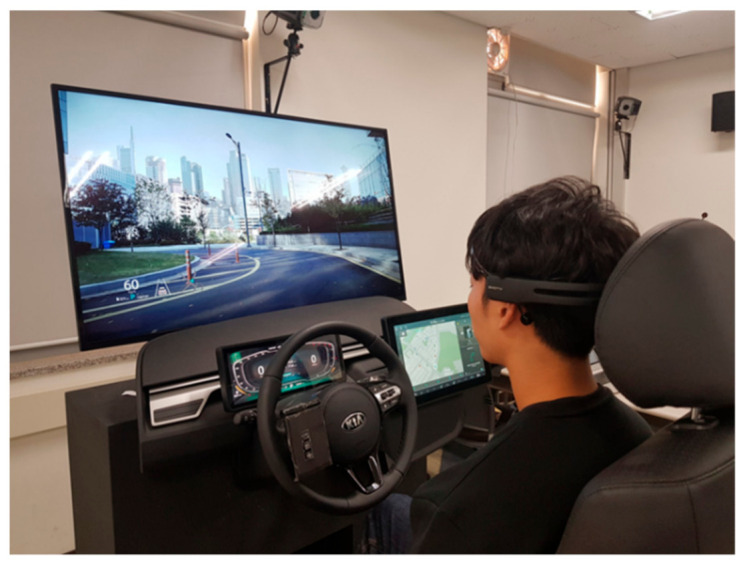
Simulator and displays.

Participants performed the tasks under four display conditions: two were based on display orientation or axis (horizontal or vertical) and the other two were based on cluster location (right side: right side of the driver or centre of the vehicle, or front: in front of the driver, which is the traditional cluster location). The participants were briefed on the task procedure by the experimental facilitator. For the display type with a cluster at the right-side location, participants were instructed to respond through the same screen, while for the cluster position at the front, they had to respond with a button at the simulator handle. Participants were requested to perform the tasks assuming a driving scenario (Figure 2).

### 2.4. Procedure

The experimental process started with an explanation regarding the objective and procedure of the experiment to all participants. The participants practised the task performance to familiarize themselves with the task before the actual experiment. Before starting the actual experiment, the EEG sensor headset was calibrated according to each participant and proper working was confirmed. A 100 percent contact quality of the EEG sensor headset was also confirmed for each participant. We measured one minute of EEG signals in a normal condition, for comparison with EEG signals during the experimental task.

**Figure 3:**
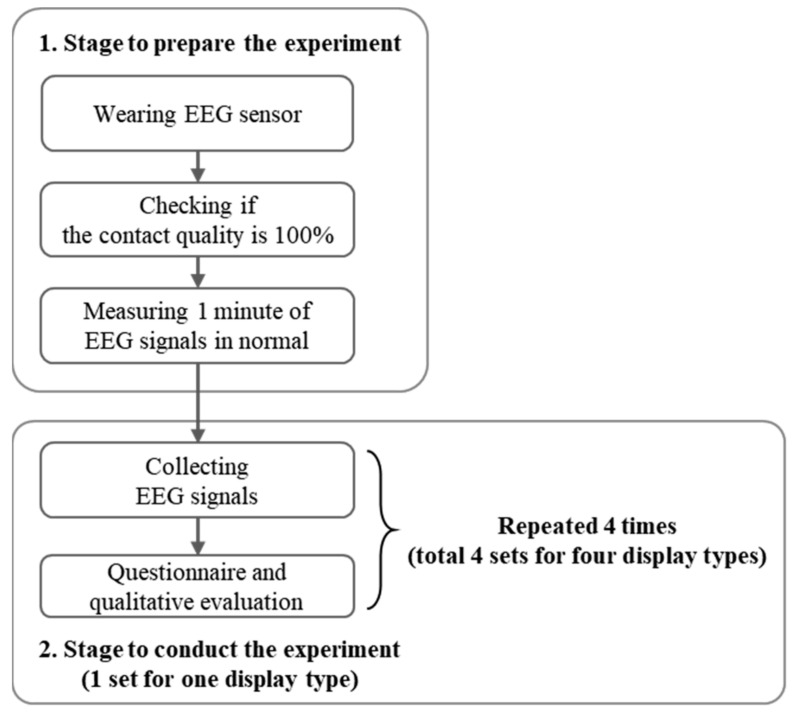
Experimental procedure.

The order of this experiment was balanced based on the balanced Latin square method. EEG signals were collected while performing the task. At the end of the experiment on each display type, the questionnaire and qualitative evaluations were collected (Figure 3). The experiment lasted approximately 2 h. A rest time of 5 min was provided between the two conditions (Figure 3).

### 2.5. Measurements

In this study, both subjective and objective measures were taken. Task completion time and performance-related psychophysiological indices were collected as objective indicators. The task completion time was defined as the time required to complete the task after the task instructions were given. This was extracted by marking the start and end moments of the task using the Tobii Pro Lab program. The psychophysiological indices were stress, engagement, interest, and relaxation, which were analysed using EEG signals in EmotivPRO [30] (Table 3). EmotivPRO preprocessed the measured raw EEG signals using a high-pass filter. The high-pass filter was based on 0.16 Hz offset electromagnetic artefacts [31]. In addition, EmotivPRO analysed the psychophysiological indices using the performance metrics function developed by Emotiv. The performance metrics function uses a machine learning algorithm for classifying and grading the intensities of various conditions, and many additional biometric measures (heart rate, respiration, blood pressure, blood volume flow, skin impedance and eye tracking) were used to develop the algorithm. The algorithm was observed and recorded by a trained psychologist, and self-reported data were also used [32]. The performance metrics function provides cognitive states in real time, such as stress, engagement, interest, and relaxation. Additionally, responses to questionnaire items in the NASA-TLX, reduced clutter score (RCS), driving activity load index (DALI), and driving experience (DX) questionnaires were also collected as subjective measures. The items of each questionnaire were evaluated on a 100-point scale (Table 4).

### 2.6. Data Analysis

A model that estimates objective indicators (i.e., performance-related psychophysiological indices) was analysed using subjective questionnaire items related to in-vehicle display usability. For this purpose, a stepwise regression analysis was performed using the bidirectional elimination method. Questionnaire data collected subjectively were used as independent variables, whereas objective indicators from the EEG data were used as dependent variables. The objective indicators were four psychophysiological indices (Table 3), and the subjective indicators were 21 subjective questionnaire items (Table 4). In addition, the averages of the performance-related psychophysiological indices over the four display conditions were confirmed. The EEG data as the objective data and the questionnaire data as the subjective data from four participants were used.

Additionally, the effect of the display condition on task performance and the subjective indicators was analysed using the data set of questionnaire items. A one-way repeated-measures ANOVA was performed to confirm statistical significance. The dependent variables were task completion time, overall workload, overall usability, and overall satisfaction. The independent variables were the display conditions (four conditions based on the cluster location and axis orientation). A Student–Newman–Keuls test was performed for post hoc analysis. A *p*-value of less than 0.05 was set as the criterion for significance.

## 3. Results

### 3.1. Model

As a result of the modelling, engagement had 91% explanatory power, and the model comprised hedonic quality, pragmatic quality, auditory demand, and memorability. Stress had 78% explanatory power, and a model comprising colourfulness and hedonic quality was derived. Relaxation had 64% explanatory power, and it was confirmed that it was related to colourfulness, physical demand, and effort. Interest was found to have 14% explanatory power and was based on hedonic quality (Table 5). Note that due to the small number of participants, this model may have limitations.

### 3.2. Task Completion Time

**Figure 4:**
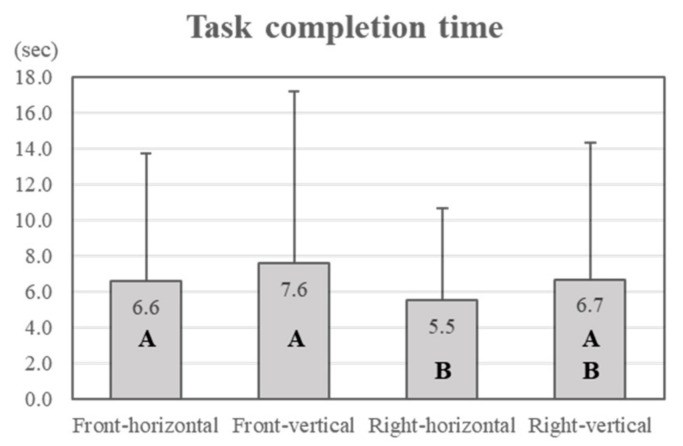
Task completion time (different letters in the bar graph indicate statistical differences).

The task completion time was analysed according to the four display conditions. There was a statistically significant difference (*p* = 0.000; α = 0.05) between task completion times for the different display conditions. In Figure 4, the bar graphs show the means of the task completion times and error bars indicate standard deviations. The task completion time was the shortest for the right–horizontal display (5.5 ± 5.1 s) and longest for the front–vertical display (7.6 ± 9.6 s).

### 3.3. Subjective Questionnaire

**Figure 5:**
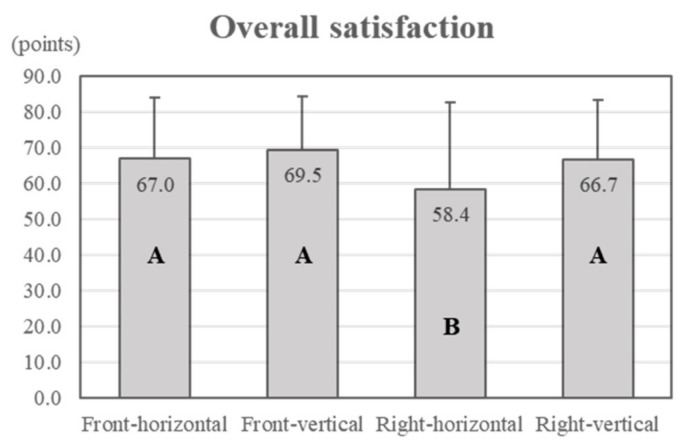
Overall satisfaction (different letters in the bar graph indicate statistical differences).

The driver’s evaluations of overall workload, usability, and satisfaction with their experience were confirmed according to the display type. Hence, the subjective questionnaire items of overall workload, overall usability, and overall satisfaction were analysed. From the analysis, overall workload and overall usability were not statistically significant (overall workload, *p* = 0.092; overall usability, *p* = 0.150; α = 0.05). Conversely, overall satisfaction was statistically significant (*p* = 0.014; α = 0.05). According to the post hoc results, front–horizontal, front–vertical, and right–vertical were found to form a group with no statistically significant differences in overall satisfaction. The right–horizontal display was statistically independent (Figure 5). Overall satisfaction was highest for the front–vertical display (69.5 ± 14.9 points) and lowest for the right–horizontal display (58.4 ± 24.3 points).

## 4. Discussion

### 4.1. Performance-Related Psychophysiological Indices

According to our results, it was confirmed that subjective questionnaire items can predict objective performance-related psychophysiological indices. Additionally, subjective indicators that describe objective indicators were analysed through modelling. Previous studies have shown that EEG signals can predict a driver’s cognitive condition [19,39,40]. In previous studies, modelling analyses were performed to propose a model that recognizes the driver’s condition; however, there is insufficient research on the prediction of performance-related psychophysiological indices through subjective indicators. In this study, objective and subjective data were evaluated in driving conditions, and the relationship between them was shown.

In this study, performance-related psychophysiological indices based on EEG signals were extracted as objective indicators. Holman and Adebesin [41] analysed performance-related psychophysiological indices extracted using EEGs for user experience (UX). According to the results, the indicators objectively evaluated UX. This study adopted the same modelling approach to analyse the relationship for subjective questionnaire items. As a result, models with 14–91% explanatory power were derived. Therefore, this study suggested the possibility of analysing a predictive model for each psychophysiological index, as a preliminary study, even though the number of participants was small. In addition, a framework that can be applied to other studies was provided. Therefore, this study has contributed to the analysis of the relationship between electroencephalograms and subjective measurements.

Task performance using the in-vehicle display simultaneously with the main task of driving results in safety issues [1,2]. Engagement is an indicator of the degree to which the driver checks driving-related information and focuses on the task while operating the AVN. This metric is important because it allows us to determine the extent to which the driver is focusing on secondary tasks. If the engagement is small, it can be considered that the performance of the task is low; however, it is estimated that it can act as a load even if engagement is too large. Research has shown that engagement is influenced by the degree to which the vehicle’s interface is enjoyable, practical, and intuitively easy to understand, and this appears to be associated with the auditory demands during driving activities. Conversely, stress is an index that can confirm the difficulty of a task. It indicates the degree to which any display positively (+) or negatively (−) affects pleasure or mental health during driving.

Relaxation is an index that can confirm the degree to which the driver is likely to lose interest in the task after using the vehicle display. It was confirmed that relaxation is affected by the degree of colour variation in the display and the degree to which physical activity is required. In particular, it was found that the level of effort required to perform the task successfully had a negative (−) effect. Therefore, it is important to make the driver feel that less effort is required for in-vehicle display tasks, as this can increase the focus on driving. We also saw the latent effect of pleasure on interest during driving. Interest is an indicator of the degree to which the driver is attracted to the AVN operation.

### 4.2. Objective and Subjective Indicators

According to the results for task completion time, the participants took longer to complete tasks on display types with cluster locations at the front. In terms of display orientation, task completion time was longer for vertical displays than for horizontal displays (Figure 4). Therefore, the right–horizontal type of display is recommended for fast task performance, in terms of safety in this study.

Conversely, satisfaction showed different results from the task completion time. It was higher for the front–vertical display than the front–horizontal display; however, it was lower for right-side displays than for front displays. In particular, the right–horizontal display showed the lowest score compared to other display types in terms of overall satisfaction. In addition, the participants’ responses showed that the front display with a vertical axis, which required the longest time to perform the task, was the most satisfying. These results show that other factors may exist that affect the participants’ satisfaction, apart from fast task completion.

Therefore, considering low task completion time and high satisfaction at the same time, this study recommends the right-side display with a vertical axis. In future studies, it is thought that additional research on display user interaction (UI) should be performed to increase driver satisfaction.

### 4.3. Qualitative Evaluation

According to the qualitative data for each display type, some subjects responded that they felt awkward and anxious in the task because they were unfamiliar with the placing of the existing cluster on the right-side display. Furthermore, some subjects stated that the size of the existing display was too large, therefore, the function was not visible at a glance. On the contrary, the right-side display has the advantage of checking at a glance because the overall size of the display operation area is reduced owing to the inclusion of clusters in the screen.

Many subjects thought that the horizontal display was too long and wide, and they evaluated it as uncomfortable as they had to move their body to perform tasks. Some of the subjects even stated that they were concerned about their safety while driving. In particular, opinions were collected from female subjects saying, “I do not wish to use it at all” and “It does not seem attractive to me”. Conversely, some subjects had difficulty recognizing information because they were not familiar with checking information on a vertical screen. Therefore, to increase the driver’s satisfaction, it is considered that the task area of the display should be located close to the driver.

### 4.4. Limitation

In this experiment, EEG data were collected from four participants. The small number of subjects is a limitation, as it is not sufficient to generalize the experimental results. However, the same experiment was conducted with 32 subjects to confirm whether the subjective indicators were valid. In addition, we confirmed the possibility of predicting a driver’s cognitive performance using EEG indicators. The tasks were performed in an experimental environment using a driving simulator, not in an actual driving situation. In this experiment, driving conditions were assumed and participants were requested to perform the experiment; however, it seems that the experimental environment was insufficient to manipulate the display while considering the actual driving situation. This experiment took a long time and was repeated using a similar task. Therefore, there is a limitation in that the participant may feel bored or irritable, which may affect the EEG signal. To minimize this, participants were allowed to rest at the end of each set of experiments.

Due to the small number of experimental participants, it is difficult to generalize the results of this study. In other words, it is impossible to attach great significance to the numbers derived from the model. Therefore, further studies should recruit sufficient participants to draw statistically significant conclusions. Although there are limitations, this study laid the foundations for other studies as a method of analysis for the relationship between a subject’s evaluation and EEG signals, as a preliminary study.

## 5. Conclusions

In this study, the relationship between objective and subjective indicators to evaluate usability was analysed using stepwise regression analysis. To this end, the usability of various in-vehicle displays was evaluated as a case study, and the participants performed tasks using the IVISs. Performance-related psychophysiological indices and task completion times were collected as objective indicators, and questionnaire items were collected as subjective indicators. Stepwise regression was performed to confirm the relationship between psychophysiological indices and questionnaire items. The analysis revealed that the predictive model for each psychophysiological index could have a high explanatory power of up to 91%, although the number of participants was limited. Questionnaire items related to each indicator were also identified. In addition, the results for task completion time and overall satisfaction were compared and analysed for the four displays. As a result, this study recommends presenting the cluster screen together with the AVN screen on the right side (centre of vehicle) of the driver along the vertical axis. The drivers performed well with the display but subjectively felt unsatisfied. To improve this, additional research on the UI of the display should be performed. The results of this study can be used as basic data for research on objective and subjective indicators and the relationship between them. In addition, it is expected that the study can be used as reference material in research for evaluating the usability of in-vehicle displays.

## Figures and Tables

**Table 1:** Four types of displays and their attributes.

Displays	Cluster Location	Axis of Display
Front–horizontal	Front side (monitor behind handle)	Horizontal
Front–vertical	Front side (monitor behind handle)	Vertical
Right–horizontal	Right side (centre fascia monitor)	Horizontal
Right–vertical	Right side (centre fascia monitor)	Vertical

**Table 2:** Experimental tasks.

Task	Function	Instruction
Task 1	Radio	Play the FM 107.7 MHz channel on the radio.
Task 2		Save FM 107.7 as a pre-set and delete the saved channel FM 93.9.
Task 3	Calling	Find Oh Kyung-ah’s mobile phone number and call.
Task 4	Vehicle status	Set the driver’s seat to 23° and the assistant’s seat to 18°.
Task 5	Vehicle status	Set the air volume to the strongest setting.
Task 6	Calling	Find Oh Kyung-ah in the integrated favourites.
Task 7	Vehicle Status	Close the door of the passenger seat.
Task 8	Navigation	Set Jamsil Baseball Stadium as your destination.
Task 9		Change the centre of cluster screen to navigation (for cluster). Change cluster screen in AVN to driving assist state (for clusterless).
Task 10	Calling	Reject incoming calls on AVN screen.
Task 11	Advanced smart cruise control (ASCC)	Set the ASCC to speed 80 and distance between cars in 2 steps on the AVN screen.
Task 12		Change the centre screen from the cluster screen to the ASCC screen (for cluster).
Task 13	Tyre pressure monitoring system (TPMS)	Check TPMS through AVN screen.
Task 14		Check the TPMS on the cluster screen (for cluster).

**Table 3:** Definitions of psychophysiological indices.

Index	Definition
Stress	Measurement of the level of difficulty with the current challenge
Engagement	Level of attention and concentration in the moment
Interest	Degree of attraction to the current stimuli, environment, or activity
Relaxation	Measurement of the ability to switch off from intense concentration

**Table 4:** Questionnaire items.

Questionnaire	Questionnaire Item	Definition	Reference
NASA-TLX	Mental demand	Level of mental and cognitive burden	Hart and Staveland [33]
	Physical demand	Degree to which physical activity is required	
	Temporal demand	Degree to which time pressure is felt	
	Effort	Level of effort made to achieve the tasks successfully	
	Performance	The extent to which the task result was failure or success	
	Frustration	Comprehensive degree of insecurity, frustration, and anger in tasks	
	Overall workload	Overall workload from driving and vehicle-related tasks	
RCS	Overall clutter	Degree to which information presented is generally distracting and complex	Kaber et al. [34]
	Variability	How often information is displayed and how dynamic it is	
	Consistency	Degree of inconsistency in how information is presented	
	Colourfulness	How many colours are used to display information	
DALI	Visual demand	Visual demand for driving activities	Pauzié [35]
	Auditory demand	Audible demands for driving activities	
	Interference	Degree to which tasks that are not related to driving (e.g., pressing a button) are disturbed	
DX	Hedonic quality	Degree to which pleasure is obtained from the in-vehicle interface	Schwarz and Fastenmeier [36] Chi and Dewi [37] Francois et al. [38]
	Pragmatic quality	Degree to which the in-vehicle interface is practical	
	Familiarity	Degree to which the in-vehicle interface is familiar for performing the task	
	Learnability	Degree to which it is easy to learn to familiarize yourself with the vehicle’s interface	
	Memorability	Degree to which the vehicle interface is intuitively easy to understand	
	Overall usability	Degree to which the vehicle interface is easy to use overall	
	Overall satisfaction	Overall satisfaction with the vehicle interface	

**Table 5:** Modelling results for psychophysiological indices.

Indices	Adj. R^2^	Detailed Model
Stress	0.78	−0.04 + 0.04 × (Colourfulness) − 0.01 × (Hedonic quality)
Engagement	0.91	0.71 − 0.02 × (Hedonic quality) + 0.01 × (Pragmatic quality) + 0.01 × (Auditory demand) + 0.01 × (Memorability)
Interest	0.14	1.01 + 0.002 × (Hedonic quality)
Relaxation	0.64	−0.95 + 0.04 × (Colourfulness) + 0.03 × (Physical demand) − 0.02 × (Effort)

Adj. R^2^ indicates the adjusted R^2^.

## Data Availability

The data presented in this study are available on request from the corresponding author.
